# A Review of Rumen Biohydrogenation and Efficient, Green Production of High-Quality Ruminant Products: Mechanisms, Opportunities and Challenges

**DOI:** 10.3390/ani16152424

**Published:** 2026-08-05

**Authors:** Zixin Guan, Zhiyuan Ma, Fei Li, Xiumin Zhang, Li Wang, Huimin Li, Wei Zhang, Hui Xu

**Affiliations:** 1 State Key Laboratory of Herbage Improvement and Grassland Agro-Ecosystems, College of Pastoral Agriculture Science and Technology, Lanzhou University, Lanzhou 730020, China; guanzx2025@lzu.edu.cn (Z.G.); lfei@lzu.edu.cn (F.L.); 120220900671@lzu.edu.cn (L.W.); 2 State Key Laboratory of Forage Breeding-by-Design and Utilization, Key Laboratory for Agro-Ecological Processes in Subtropical Region, Institute of Subtropical Agriculture, Chinese Academy of Sciences, Changsha 410125, China; xmzhang@isa.ac.cn; 3 Minqin County Defu Agricultural Technology Co., Ltd., Minqin 733399, China; lihm0301@hotmail.com (H.L.); zhangw0408@hotmail.com (W.Z.); xuh0403@hotmail.com (H.X.)

**Keywords:** biohydrogenation, fatty acid, methane, hydrogen, rumen

## Abstract

Rumen biohydrogenation is a microbial process that converts dietary unsaturated fatty acids into more saturated products and produces a range of intermediates that can affect animal products and rumen fermentation. This review summarizes current knowledge on the main biohydrogenation pathways, the microbial groups involved, and the nutritional and breeding strategies used to regulate this process. Particular attention is given to the transformation of C18 fatty acids, the formation of vaccenic acid and conjugated linoleic acid, the trans-10 shift, and the links between lipid metabolism, hydrogen use, and methane formation. Evidence indicates that diet composition, lipid source, plant secondary metabolites, rumen-protected fat technologies, microbial interventions, and host-related factors can all influence biohydrogenation outcomes. These strategies can improve the fatty acid profile of meat and milk, but their effects are context-dependent and vary with animal species, diet, and rumen microbial structure. Important gaps remain, including the identification of active microbial populations, the functions of many transient intermediates, and the extent to which changes in biohydrogenation directly contribute to methane mitigation. Clarifying these mechanisms is essential for improving the nutritional quality of ruminant products and the sustainability of production systems.

## Simple Summary

Meat and milk from cattle, sheep, and goats provide important nutrients, but the types of fat they contain are strongly influenced by microorganisms in the rumen, the first stomach compartment. These microorganisms convert many dietary unsaturated fats into more saturated forms and produce intermediate compounds that can affect food quality, methane formation, and milk fat production. This review explains the main pathways involved, the microorganisms that contribute, and the feeding, microbial, technological, and breeding strategies that may modify these changes. Current evidence shows that diet composition, fat source, plant compounds, fats designed to avoid breakdown in the rumen, and animal-related factors can improve the types and proportions of fats in meat and milk, but responses vary among animals and feeding conditions. This fat-conversion process uses only a small share of the hydrogen released during rumen fermentation, so its direct contribution to methane reduction is limited. Some methane-reducing diets may also increase the risk of reduced milk fat production. Progress will require experiments that directly link diet, rumen microbial activity, fatty acid conversion, and animal responses. Such evidence could support more precise feeding strategies for producing healthier ruminant foods with lower environmental costs.

## 1. Introduction

Growing awareness of the relationship between diet and health has increased consumer interest in high-quality animal products with improved nutritional value. In ruminant products, the fatty acid (FA) profile is an important determinant of nutritional quality. Higher proportions of n-3 polyunsaturated fatty acids (PUFA), conjugated linoleic acid (CLA), and vaccenic acid (VA) have been associated with potential health-related benefits, including anti-inflammatory, cardioprotective, and immune-related effects [1,2].

Improving the FA composition of ruminant-derived foods has therefore become an important goal in animal nutrition research. Rumen biohydrogenation (BH), a microbial process that converts dietary unsaturated fatty acids (UFA) into more saturated products and generates bioactive intermediates, plays a central role in shaping the FA profile of meat and milk [1,3]. At the same time, rumen lipid metabolism is connected with metabolic hydrogen use and methanogenesis, linking product quality with methane emissions and the environmental performance of ruminant production systems [3,4,5].

This review examines the mechanisms, microbial contributors, and regulatory approaches of rumen biohydrogenation, with emphasis on two unresolved questions: when changes in biohydrogenation contribute directly to methane emissions and product fatty acid quality, and when these outcomes arise independently from broader changes in diet and rumen fermentation. On this basis, we compare the strength of the available evidence, methodological limitations, and intervention-specific trade-offs across ruminant production systems.

## 2. Methods

This narrative review was based on searches of the Web of Science Core Collection, with the final search conducted on 5 July 2026. The main searches, conducted using the TS (Topic) field, were: TS = (“rumen biohydrogenation” OR “ruminal biohydrogenation”), which retrieved 797 records; TS = (rumen OR ruminal) AND TS = (“fatty acid” OR “fatty acids” OR lipid OR lipids), which retrieved 13,554 records; TS = (“rumen biohydrogenation” OR “ruminal biohydrogenation”) AND TS = (milk OR meat OR “fatty acid profile” OR “product quality”), which retrieved 626 records; TS = (“rumen biohydrogenation” OR “ruminal biohydrogenation”) AND TS = (methane OR methanogenesis OR hydrogen), which retrieved 179 records; TS = (“rumen biohydrogenation” OR “ruminal biohydrogenation”) AND TS = (microbiota OR microbiome OR bacteria OR microbial), which retrieved 325 records; and TS = (“rumen biohydrogenation” OR “ruminal biohydrogenation”) AND TS = (diet OR nutrition OR tannin OR “plant secondary metabolites” OR “protected fat” OR “lipid supplementation”), which retrieved 639 records.

Records were screened mainly by title and abstract. Studies were retained when they addressed rumen biohydrogenation, ruminal lipid metabolism, fatty acid composition of meat or milk, microbial involvement, methane-related hydrogen metabolism, or nutritional regulation. Studies involving non-ruminants, human nutrition without a rumen context, unrelated topics, or duplicated information were excluded. Peer-reviewed research articles and reviews with clear mechanistic or production relevance were prioritized.

**Figure 1:**
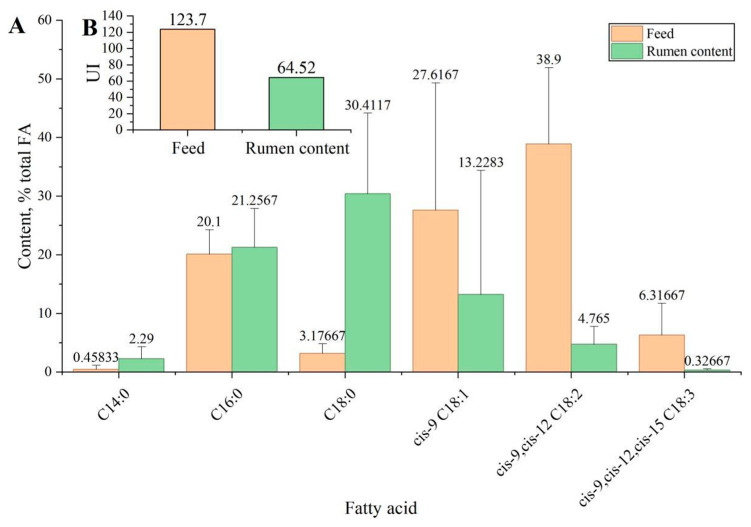
Descriptive comparison of fatty acid composition and unsaturation between feed and rumen contents. Panel (**A**) shows the unweighted arithmetic mean concentrations of six major fatty acids in feed and the corresponding rumen contents across six paired datasets; error bars represent standard deviations. Panel (**B**) shows the mean unsaturation index of feed and rumen contents. The unsaturation index was calculated as the sum of the percentage of each fatty acid multiplied by its number of double bonds. No inferential statistical testing was performed. Because the included studies differed in animal species, diets, sampling procedures, and analytical methods, the values represent overall descriptive trends rather than pooled effect estimates. The paired fatty acid data and summary statistics for Panel (**A**) are provided in Table S1A, whereas the paired unsaturation index data for Panel (**B**) are provided in Table S1B.

For Figure 1, six paired feed–rumen datasets from four publications were compiled [4,6,7,8]. Major fatty acids were summarized as unweighted arithmetic means with standard deviations, whereas the unsaturation index was summarized as an unweighted arithmetic mean. The unsaturation index was calculated as the sum of the percentage of each fatty acid multiplied by its number of double bonds. Figure 1 was generated using OriginPro 2026 (OriginLab Corporation, Northampton, MA, USA), and no inferential statistical analysis was performed. The paired fatty acid data and summary statistics used for Figure 1A are provided in Table S1A, whereas the paired unsaturation index data used for Figure 1B are provided in Table S1B.

**Figure 2:**
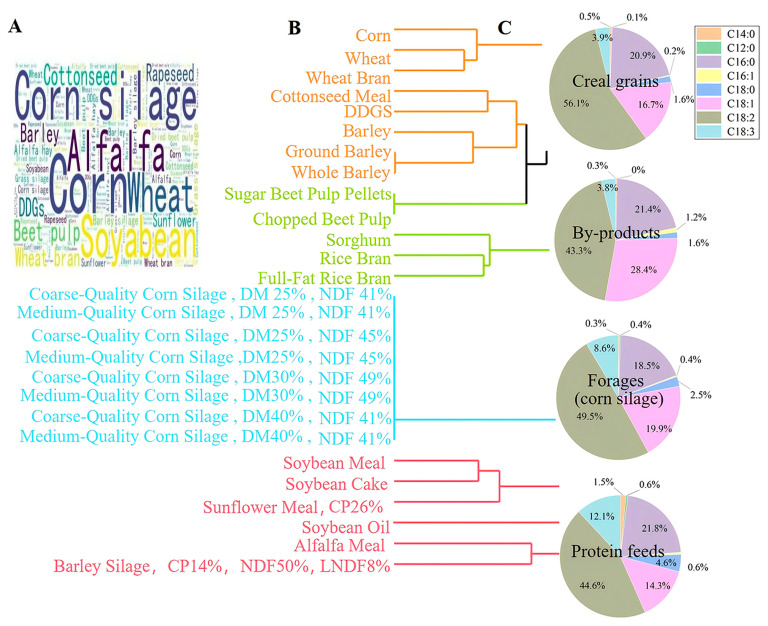
Commonly used feed ingredients in ruminant diets and their fatty acid characteristics. Panel (**A**) presents a descriptive word cloud based on the occurrence frequencies of standardized feed ingredients, with word size proportional to occurrence frequency. Panel (**B**) shows a three-cluster hierarchical analysis of feed ingredients based on ether extract and eight fatty acid variables after data standardization using Ward’s method in JMP Pro 16. Panel (**C**) presents independently generated 2D pie charts of the fatty acid composition of four predefined feed categories: cereal grains, by-products, forages (corn silage), and protein feeds; these charts were generated using OriginPro 2026. The three panels were generated independently and assembled into a single composite figure. The underlying data are provided in Tables S2a, S2bA, and S2bB, respectively.

For Figure 2A, feed-ingredient names were standardized before their occurrence frequencies were counted, and word size was proportional to frequency. For Figure 2B, hierarchical clustering was performed in JMP Pro 16 (JMP Statistical Discovery LLC, Cary, NC, USA) using ether extract and eight fatty acid variables. Variables were standardized, Ward’s method was applied, and three clusters were retained. Figure 2C was generated independently in OriginPro 2026 using four predefined feed categories. The corresponding data are provided in Tables S2a, S2bA, and S2bB.

**Figure 3:**
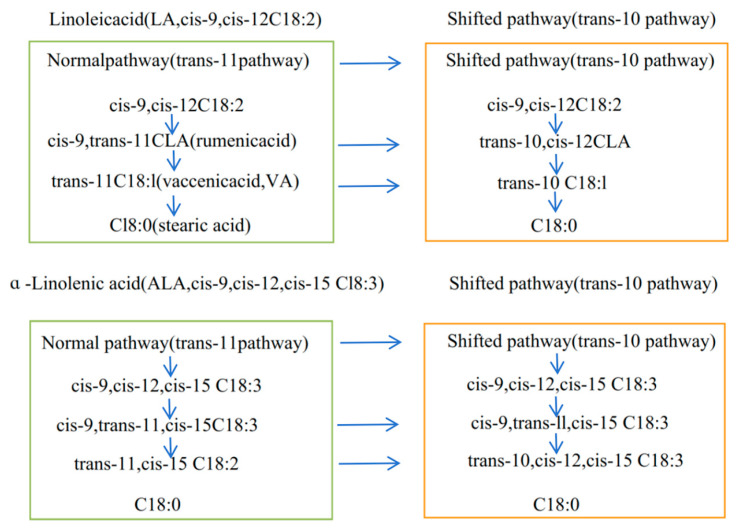
Main rumen biohydrogenation pathways of dietary C18 polyunsaturated fatty acids. The left side summarizes the conventional trans-11 pathway, in which linoleic acid and alpha-linolenic acid are converted to stearic acid through intermediate products. The right side shows the alternative trans-10 pathway, which has been associated with high-starch diets, low rumen pH, or a high unsaturated fatty acid load, and with the accumulation of trans-10 intermediates. Vertical arrows indicate successive conversion steps within each pathway, whereas horizontal arrows indicate the shift from the conventional trans-11 pathway to the alternative trans-10 pathway.

For Figure 3, the conventional trans-11 and alternative trans-10 biohydrogenation pathways were summarized from the cited literature and redrawn by the authors.

**Figure 4:**
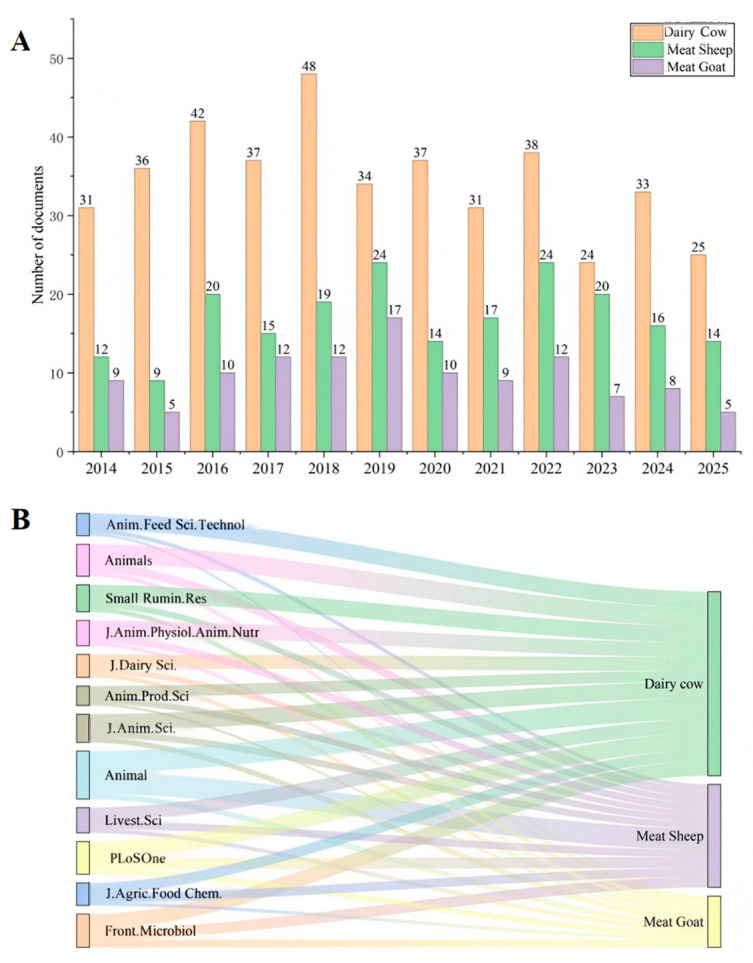
Publication patterns of rumen biohydrogenation-related studies across animal categories and journals. Panel (**A**) shows the annual numbers of records involving dairy cows, meat sheep, and meat goats from 2014 to 2025; the underlying data are provided in Table S3a. Panel (**B**) presents a Sankey diagram illustrating the relationships between journal source nodes and animal categories. The Sankey diagram was generated in OriginPro 2026 from a three-column table containing source journal node, target animal category, and flow value, with flow width proportional to the corresponding input value; the underlying data are provided in Table S3b. The two panels were generated independently and assembled into a single composite figure.

For Figure 4, bibliographic records were manually organized by publication year, journal source, and animal category. Annual counts for dairy cows, meat sheep, and meat goats from 2014 to 2025 were plotted in OriginPro 2026. The Sankey diagram was generated separately from a three-column table containing source journal, target animal category, and flow value. The underlying data are provided in Tables S3a and S3b. All figures were used for descriptive synthesis rather than meta-analysis.

## 3. Rumen BH Pathways

### 3.1. Concept of Rumen BH

Rumen biohydrogenation is generally regarded as a microbial detoxification process that reduces the antimicrobial effects of dietary unsaturated fatty acids. It is mainly carried out by rumen bacteria, with anaerobic fungi making a smaller but still relevant contribution. The process involves isomerization, sequential hydrogenation, double-bond migration, hydration, and oxidation reactions, through which unsaturated fatty acids are progressively converted into more saturated products. For most C18 unsaturated fatty acids, stearic acid is the major end product [9,10].

To compare the extent of rumen biohydrogenation across studies, we integrated data from published reports that provided fatty acid profiles of both feed and rumen contents. The unsaturation index was used to describe the overall degree of fatty acid unsaturation [11].

Across the selected datasets, the unsaturation index decreased from 123.70 in the diet to 64.52 in rumen digesta, indicating extensive hydrogenation of dietary unsaturated fatty acids in the rumen (Figure 1). Among the major fatty acids, C18:0 showed the largest increase from feed to rumen contents, whereas C16:0 changed only slightly. This pattern supports the view that C18 unsaturated fatty acids are the main substrates for rumen biohydrogenation, while C16 fatty acids contribute less to the overall process, partly because C16 unsaturated fatty acids are usually present at lower levels in ruminant diets (Figure 1A; Table S1A). However, because the selected studies differed in animal species, diet composition, sampling procedures, and analytical methods, these integrated values should be interpreted as an overall trend rather than a pooled quantitative effect.

The composition of C18 fatty acids can therefore serve as an indicator of rumen biohydrogenation activity. In addition to the unsaturation index, biohydrogenation completeness has been proposed to describe the proportion of C18:0 actually produced relative to the theoretical amount that would be obtained if all dietary C18 unsaturated fatty acids were fully hydrogenated [12,13]. The relative abundance of specific intermediates is also informative. For example, the trans-10/trans-11 ratio is commonly used to evaluate shifts in biohydrogenation pathways, especially the occurrence of a trans-10 shift associated with less favorable fatty acid profiles [12]. Plasma fatty acid ratios may also provide a non-invasive way to monitor changes in rumen biohydrogenation status [12].

### 3.2. Rumen BH of C18:2 PUFA

In the rumen, the BH of C18:2 involves a sequence of tightly regulated microbial reactions, including isomerization, stepwise hydrogenation, and alternative metabolic routes [9]. The major PUFA in the diet is linoleic acid (Figure 2).

Linoleic acid, primarily present in feedstuffs as part of triacylglycerols, is initially isomerized to cis-9,trans-11 CLA (Figure 3), a bioactive intermediate with recognized health benefits [9]. This CLA is subsequently reduced to trans-11 C18:1, which is finally hydrogenated to C18:0 [3,9]. This sequence is commonly referred to as the trans-11 pathway and is considered the canonical BH route. However, under conditions of high dietary starch or high rumen UFA load, the BH pathway can shift toward an alternative route known as the trans-10 shift [10]. In this alternative pathway, linoleic acid is converted to trans-10,cis-12 CLA and subsequently to trans-10 C18:1. Trans-10,cis-12 CLA is a potent inhibitor of mammary lipogenesis, whereas trans-10 C18:1 is commonly regarded as a marker of the trans-10 shift and is associated with less favorable milk- and meat-fat profiles [10,14]. However, high-starch diets or elevated UFA supply do not consistently induce a trans-10 shift. This inconsistency indicates that carbohydrate fermentability, effective fiber, adaptation period, and the initial microbial community also influence the response.

### 3.3. Rumen BH of n-3 PUFA

Ruminant diets can supply several n-3 polyunsaturated fatty acids, including eicosapentaenoic acid (EPA), docosapentaenoic acid (DPA), and docosahexaenoic acid (DHA) [15]. These long-chain n-3 fatty acids are mainly supplied by marine sources and microalgae. Endogenous synthesis from alpha-linolenic acid is possible, but this conversion is limited. Therefore, the amount of long-chain n-3 fatty acids available for absorption depends on both dietary supply and the extent of rumen metabolism.

Alpha-linolenic acid undergoes extensive biohydrogenation in the rumen. It is first converted into a conjugated triene intermediate, cis-9,trans-11,cis-15 C18:3, and then reduced to trans-11,cis-15 C18:2. This intermediate can enter the vaccenic acid pool before being further hydrogenated to C18:0. Because alpha-linolenic acid contains more double bonds than linoleic acid, it is usually hydrogenated more rapidly and more extensively. However, its pathway is still sensitive to dietary composition, rumen pH, and microbial shifts [3,9,16].

Under high-starch diets, low rumen pH, or high unsaturated fatty acid load, alpha-linolenic acid can also be redirected from the conventional trans-11 pathway toward the trans-10 pathway [10,17]. In this route, alpha-linolenic acid is first isomerized to trans-10,cis-12,cis-15 C18:3 and then further hydrogenated to trans-10 C18:1. Because the final reduction to C18:0 is inefficient, trans-10 intermediates may accumulate in the rumen and be transferred to mammary and muscle tissues [10,17]. Previous regression analyses showed that rumen alpha-linolenic acid disappearance was positively associated with trans-10,cis-15 C18:2 formation, which was further correlated with trans-10 C18:1 accumulation [12]. For this reason, the trans-10,cis-15/trans-11,cis-15 C18:2 ratio has been proposed as a sensitive indicator of trans-10 shifted biohydrogenation [12].

Marine lipids add another layer of complexity to n-3 fatty acid metabolism in the rumen. Eicosapentaenoic acid and docosahexaenoic acid both inhibit the final hydrogenation step from trans-11 C18:1 to C18:0, leading to the accumulation of C18:1 and conjugated linoleic acid intermediates [12,15]. However, their effects are not identical. DHA promotes alternative biohydrogenation pathways more strongly, particularly the accumulation of trans-10 C18:1, whereas EPA has more specific effects on C18:3 n-3 metabolism [15]. This distinction is important because it helps explain why marine lipid supplementation can improve the n-3 fatty acid content of milk and meat while also increasing the risk of milk fat depression under certain dietary conditions.

Reported responses to marine lipids differ among ruminant species and production stages; however, direct comparisons between cows and sheep should be interpreted cautiously because the available studies also differ in lipid dose, basal diet, physiological stage, and experimental design. Comparative in vitro studies have identified species-dependent differences in the ruminal metabolism of EPA, DPA, and DHA, whereas *in vivo* fish-oil supplementation has produced variable effects on milk fat content and milk yield [15,18]. In pregnant Awassi ewes, supplementation with fish oil at 2.1% of dietary dry matter during late gestation increased gestation length and lamb birth weight, while temporarily reducing milk yield during early lactation [18]. Compared with EPA and DHA, DPA has weaker direct effects on rumen biohydrogenation and influences milk fat composition mainly through endogenous tissue metabolism [19].

In addition to hydrogenation, unsaturated fatty acids also undergo partial hydration and oxidation in the rumen. For example, cis-9 C18:1 can be converted to 10-hydroxystearic acid and subsequently oxidized to 10-oxostearic acid [15]. Although these pathways are less frequently discussed than the classical hydrogenation routes, they show that rumen lipid metabolism includes several parallel reactions. Their quantitative contribution and biological significance remain less clear and deserve further study.

## 4. Microbial Involvement and Regulation of Rumen BH

### 4.1. Major Microbial Players in Rumen BH

Rumen BH is carried out by a highly specialized and functionally diverse microbial consortium, in which bacteria play a dominant role (Table 1). Rumen BH bacteria have been classified into two functional groups: group A hydrogenate PUFA to the VA stage, and group B further reduce monounsaturated fatty acids (MUFA) to C18:0 [3,20]. Members of the genus Butyrivibrio are among the best-characterized biohydrogenating bacteria in the rumen [3]. *Butyrivibrio fibrisolvens* and *Pseudobutyrivibrio* spp. are typically assigned to group A, whereas *Butyrivibrio proteoclasticus*—reclassified from *Clostridium proteoclasticum*—is currently the only well-characterized group B bacterium capable of catalyzing the final hydrogenation step from VA to C18:0 [3,21]. Notably, group B bacteria are highly sensitive to the toxic effects of UFA, which makes this terminal step particularly susceptible to dietary and environmental modulation [3,22]. EPA and DHA exhibit strong disruptive effects on rumen BH by competitively inhibiting key reactions or exerting toxic effects on group B bacteria, thereby blocking the rate-limiting step converting trans-FA intermediates to C18:0 and causing the accumulation of BH intermediates [3,23]. *Cutibacterium acnes* isomerizes linoleic acid to trans-10,cis-12 CLA in pure culture and may contribute to the trans-10 pathway. However, the *in vivo* trans-10 shift is likely mediated by a broader microbial consortium and is strongly influenced by rumen conditions. Trans-10,cis-12 CLA is a potent inhibitor of mammary lipogenesis, and *Butyrivibrio* spp. can reduce this intermediate to trans-10 C18:1 [10].

Anaerobic rumen fungi, including *Orpinomyces*, *Piromyces*, and *Neocallimastix*, can biohydrogenate unsaturated fatty acids, although their contribution is limited by their relatively slow growth rates (approximately 24 h life cycles) compared with bacteria (approximately 100 min) [24]. Fungal BH typically terminates at the MUFA stage, particularly VA [24]. In contrast, rumen protozoa do not synthesize BH enzymes themselves; the high concentrations of CLA and VA detected in protozoal cells are mainly attributed to the ingestion and sequestration of chloroplasts and associated BH bacteria [3]. Overall, evidence for microbial involvement in BH is uneven. The functions of several cultured strains have been experimentally demonstrated, whereas the roles of many uncultured taxa are inferred mainly from associations with fatty acid profiles and require direct functional validation.

### 4.2. Regulation of Rumen BH

Rumen biohydrogenation is strongly influenced by diet because dietary composition affects substrate supply, rumen pH, fermentation pattern, and microbial community structure. The regulatory strategies summarized in Table 2 can be grouped according to their main mode of action, including changes in lipid substrate availability, stabilization of rumen fermentation, selective inhibition of microbial groups, protection of unsaturated fatty acids from rumen metabolism, and host-related regulation.

Dietary structure is one of the main drivers of biohydrogenation pathways. High-forage diets generally favor the trans-11 pathway, whereas high-starch or high-concentrate diets increase the risk of a trans-10 shift [10,12,17]. Rapidly fermentable grains such as barley and wheat are more likely to promote trans-10 intermediates than more slowly fermentable grains such as corn [14,36]. Replacing part of the cereal grain with agro-industrial by-products rich in soluble fiber or pectin, such as beet pulp, citrus pulp, and almond hulls, can reduce starch load, stabilize rumen fermentation, and alleviate the trans-10 shift [14,26]. These effects suggest that carbohydrate source and rumen fermentation stability are as important as lipid source in determining biohydrogenation outcomes.

Lipid supplementation is a direct way to manipulate rumen biohydrogenation. Oils rich in linoleic acid or alpha-linolenic acid increase the supply of unsaturated fatty acid substrates and usually intensify biohydrogenation activity. However, the final effect depends on lipid type, dose, processing method, and rumen conditions. Processing methods such as extrusion can improve lipid availability to the host, whereas rumen-protection approaches, including calcium salts, fatty acid amides, encapsulation, and freeze-drying, aim to limit ruminal hydrogenation and increase the intestinal flow of unsaturated fatty acids [1,13]. Thus, lipid strategies must balance two goals: promoting beneficial intermediates in the rumen and avoiding excessive loss of dietary unsaturated fatty acids before absorption.

Plant secondary metabolites provide another important route for regulating biohydrogenation. Polyphenols and tannins can inhibit specific microbial steps, especially the final reduction of vaccenic acid to C18:0, which may increase the accumulation of vaccenic acid and conjugated linoleic acid in ruminant products [20,26]. However, this effect is not always beneficial. Excessive tannin supplementation, particularly in high-concentrate or high-fat diets, may increase the risk of a trans-10 shift [12,14]. Essential oils such as cinnamaldehyde, carvacrol, and thymol have antimicrobial effects and may partially protect polyunsaturated fatty acids from complete saturation [21,31]. Forages rich in polyphenol oxidase, such as red clover, can reduce initial lipolysis and indirectly slow subsequent biohydrogenation reactions [1]. Other compounds, including saponins and flavonoids, may act mainly through effects on protozoa and associated microbial populations [1,10].

Chemical additives and stabilizers can also influence biohydrogenation by modifying microbial activity or rumen conditions. For example, 2-hydroxy-4-(methylthio)butanoic acid and vitamin E have been reported to reduce trans-10 shift and milk fat depression, possibly by suppressing microbes involved in trans-10 intermediate formation [14]. Buffers such as sodium sesquicarbonate and potassium carbonate help maintain rumen pH and support trans-11 biohydrogenation pathways [10,14,16]. Physical and chemical lipid-protection methods, including formaldehyde-treated protein matrices, calcium salts, and fatty acid amides, reduce the availability of unsaturated fatty acids in the rumen and thereby change both the extent of biohydrogenation and the profile of trans-10 and trans-11 intermediates [9].

Taken together, these strategies show that rumen biohydrogenation cannot be regulated by lipid supplementation alone. The same lipid source may lead to different outcomes depending on the forage-to-concentrate ratio, rumen pH, microbial community, and the presence of plant or chemical modifiers. A practical regulation strategy should therefore consider the whole dietary system rather than a single additive.

### 4.3. Effects of Breeding Strategies on Rumen BH

From a host genetic perspective, approximately 27.6% of rumen core functional genes are significantly influenced by the host genome [5], providing a theoretical basis for microbiome-driven breeding strategies aimed at regulating rumen lipid metabolism. However, microbial enzymes directly involved in BH reactions and well-known BH bacteria are only weakly influenced by host genetics, exhibiting low heritability (h^2^ ≤ 0.10). Instead, host genetic control may indirectly regulate BH by controlling rumen pH, passage rate, and carbohydrate and protein synthesis [5]. In addition, host-directed genetic engineering may provide an additional route for improving the n-3 PUFA content of ruminant products, although this strategy acts largely independently of rumen BH [37,38].

## 5. BH in Relation to Sustainable and High-Quality Ruminant Production

### 5.1. Role of Rumen BH in Methane Emissions

Ruminal methanogenesis should be considered within a network of competing hydrogen sinks rather than as an isolated fermentation endpoint. Fermentative bacteria, protozoa, and anaerobic fungi release reducing equivalents mainly as molecular hydrogen and formate during carbohydrate degradation. Hydrogenotrophic methanogens consume these products and maintain a low ruminal hydrogen partial pressure, which supports continued substrate oxidation by hydrogen-producing microorganisms.

Rumen biohydrogenation also consumes reducing equivalents during the sequential reduction of unsaturated fatty acids. However, BH is estimated to account for only approximately 1–5% of metabolic hydrogen disposal, considerably less than methanogenesis [28,39]. An increase in BH therefore cannot be expected to produce a proportional decrease in methane formation, and parallel changes in the two processes should not be interpreted as evidence of direct hydrogen competition unless the overall hydrogen balance is quantified.

When methanogenesis is inhibited, reducing equivalents may be redirected toward propionate formation, microbial biomass synthesis, reductive acetogenesis, other reduced fermentation products, or gaseous hydrogen loss. In reductive acetogenesis, acetogenic bacteria reduce carbon dioxide with hydrogen through the Wood–Ljungdahl pathway to produce acetate, which can subsequently be used as an energy source by the host. This pathway is potentially beneficial because it conserves reducing equivalents in a nutritionally useful product. Nevertheless, reductive acetogenesis normally remains a secondary hydrogen sink in the adult rumen because hydrogenotrophic methanogens can use hydrogen at lower concentrations and generally outcompete acetogens under conventional ruminal conditions.

Genomic and metagenomic evidence indicates that the rumen contains diverse hydrogen-producing and hydrogen-consuming microorganisms, including organisms with acetogenic potential, but the extent to which this capacity becomes functionally active after methanogenesis is inhibited remains variable [28,39,40]. Consequently, suppressing methanogens alone does not guarantee that the spared hydrogen will be captured through reductive acetogenesis; part of it may instead accumulate as dissolved hydrogen, be eructated, or be redirected through other fermentation pathways.

Methanogen ecology further complicates the relationship between BH and methane formation. Hydrogenotrophic archaea, particularly members of *Methanobrevibacter* and related taxa, participate in syntrophic associations with fibrolytic bacteria, protozoa, and anaerobic fungi. Interspecies transfer of hydrogen or formate enables methanogens to obtain reducing equivalents directly from neighboring fermentative microorganisms, while hydrogen removal improves the energetic efficiency of the hydrogen-producing partner. Protozoa-associated methanogens may have particularly direct access to locally produced hydrogen. Therefore, an intervention that reduces methane may act not only through direct inhibition of methanogens, but also through changes in protozoal populations, fibrolytic activity, fungal metabolism, substrate fermentation, or the physical associations among these microbial groups [28,39,40]. The same dietary intervention may simultaneously affect biohydrogenating bacteria, which helps explain why methane emissions and fatty acid profiles often change together without BH necessarily being the direct cause of methane reduction.

Lipid supplementation illustrates this mechanistic overlap. In goats, corn-oil supplementation reduced methane yield by approximately 15.1% per kilogram of dry matter intake and increased hydrogen use associated with BH, while also changing fermentation pathways and the rumen microbial community [4]. However, the reduction in methane cannot be attributed exclusively to the hydrogen consumed by BH. Unsaturated fatty acids may directly inhibit methanogens or protozoa, suppress some hydrogen-producing fibrolytic microorganisms, reduce fiber digestion, and shift fermentation toward more reduced products such as propionate. Plant secondary metabolites may also reduce methane by acting on methanogens, protozoa, or their syntrophic partners while independently modifying the terminal steps of BH [20,28]. Methane inhibition and altered BH should therefore be viewed as potentially parallel consequences of a broader microbial and fermentation response rather than as an inherently causal relationship.

A further consideration is the potential trade-off between methane mitigation and milk fat synthesis. Diets containing high concentrations of rapidly fermentable starch and unsaturated fatty acids may reduce methane production by decreasing hydrogen generation or redirecting reducing equivalents away from methanogenesis. At the same time, low rumen pH and a high unsaturated fatty acid load can disrupt the conventional trans-11 BH pathway and promote a trans-10 shift. Accumulation of trans-10,cis-12 CLA and related trans-10 intermediates can inhibit mammary lipogenesis and cause milk fat depression [10,14,15]. Marine lipids may also inhibit the terminal reduction of trans-C18:1 to C18:0 and increase the accumulation of BH intermediates, meaning that a methane-reducing lipid strategy may improve some aspects of the milk fatty acid profile while simultaneously increasing the risk of milk fat depression. In contrast, targeted inhibition of methanogenesis with 3-nitrooxypropanol has produced persistent methane reductions without negatively affecting milk production, demonstrating that methane inhibition does not inherently require a trans-10 shift or impaired lactational performance [41]. The occurrence of a trade-off therefore depends on the mechanism of mitigation rather than on methane reduction itself.

Future mitigation strategies should consequently be evaluated as whole-rumen interventions. Measurements of methane output should be accompanied by dissolved hydrogen, volatile fatty acid patterns, hydrogen recovery, fiber digestibility, BH intermediates, methanogen activity, protozoal abundance, dry matter intake, and milk fat concentration and yield. Strategies that suppress methanogenesis while maintaining rumen pH, fiber digestion, and the trans-11 BH pathway are more likely to provide simultaneous environmental and product-quality benefits. Conversely, a reduction in methane accompanied by impaired digestion, hydrogen accumulation, a trans-10 shift, or milk fat depression should not be considered an unequivocal improvement in rumen energy use or production sustainability.

### 5.2. Rumen BH and the Nutritional Value of Ruminant Products

Rumen biohydrogenation determines the fatty acids available for intestinal absorption and thereby influences the fatty acid composition of meat and milk. Strategies that increase post-ruminal PUFA flow can increase the proportion of n-3 PUFA and reduce the atherogenic and thrombogenic indices of ruminant products [1,42]. Microbial additives have been investigated as potential modulators of rumen fermentation and BH. However, their effects on VA and CLA accumulation are strain-, diet-, and host-dependent, and consistent improvements in the fatty acid composition of milk or meat have not yet been demonstrated across studies [3,9].

Tannin supplementation can alter both rumen biohydrogenation and antioxidant status, but current evidence does not establish biohydrogenation as the cause of the antioxidant response. In finishing lambs, chestnut tannin extract altered rumen BH and body fatty acid composition while increasing plasma glutathione peroxidase and superoxide dismutase. Separate studies showed that chestnut tannins increased glutathione peroxidase and total antioxidant capacity and reduced malondialdehyde concentrations in blood or tissues. These changes were accompanied by lower milk malondialdehyde concentrations in dairy cows and improved oxidative stability of lamb meat [43,44,45].

## 6. Future Research Opportunities and Challenges

Current research on rumen biohydrogenation remains uneven across ruminant species. Studies published between 2014 and 2025 have focused mainly on dairy cows, whereas fewer studies have examined meat sheep and meat goats, and very limited work has been conducted in beef cattle (Figure 4). This imbalance restricts our understanding of species-specific biohydrogenation mechanisms and limits the transfer of findings from dairy systems to meat-producing ruminants. Future studies should therefore include a wider range of animal models and production conditions.

The microbial hosts and enzymes responsible for specific BH reactions remain poorly resolved. Although several cultured bacteria have been linked to known pathways, many functional genes and microbial hosts remain unidentified. Integrated multi-omics approaches, including metagenomics, metatranscriptomics, metabolomics, and lipidomics, offer useful tools for linking microbial function with fatty acid transformation. The increasing availability of rumen metagenome-assembled genomes provides a broader reference framework for identifying candidate organisms and functional genes involved in BH [46]. When combined with single-cell genomics, these approaches may help assign candidate BH functions to specific low-abundance or uncultured microorganisms that are difficult to resolve using bulk community analyses [47]. Stable-isotope tracing with labeled fatty acids could further provide direct evidence of substrate transformation and help distinguish microorganisms that actively participate in BH from those that are only correlated with fatty acid profiles [48]. However, association alone is not enough. Controlled feeding trials, microbial isolation, and functional validation remain necessary to identify the organisms that directly drive specific BH reactions.

Transient intermediates present a separate analytical problem. Specific trans fatty acids and conjugated linoleic acid isomers are present only briefly or at low concentrations. Their biological roles in ruminant physiology and human nutrition remain incompletely understood [9,49]. Progress in this area is limited by the availability of high-purity reference standards and by differences in analytical methods among studies. Standardized nomenclature, improved reporting of fatty acid isomers, and wider use of high-resolution chromatographic methods will be important for improving comparability across studies [9,49].

Methane-related studies must also separate changes in lipid transformation from other hydrogen-redirecting mechanisms. Lipid supplementation and plant secondary metabolites may reduce methane emissions and alter biohydrogenation at the same time, but these responses do not necessarily reflect a direct causal relationship. Because biohydrogenation accounts for only a small proportion of hydrogen use compared with methanogenesis, future studies should separate lipid transformation from other methane-reducing mechanisms, such as inhibition of methanogens, changes in protozoal populations, and shifts in fermentation pathways.

For practical application, microbiome-based indicators must be converted into reliable breeding and feeding tools. Low-cost phenotyping methods, such as infrared spectroscopy-derived proxies or blood-based biomarkers, may help apply mechanistic findings at a larger scale [5,9,28]. Building on these indicators, microbiome engineering and precision nutrition could be used to regulate specific BH pathways according to diet composition, host genetics, production stage, and baseline microbial characteristics. Artificial intelligence and machine-learning models could integrate these factors with rumen fermentation, methane output, and product fatty acid profiles to predict animal- or herd-level responses to nutritional interventions. Systems-biology approaches could further evaluate how dietary or microbial interventions redistribute carbon, hydrogen, and fatty acid intermediates among rumen fermentation, methanogenesis, BH, and animal-product synthesis. The use of agro-industrial by-products, including grape pomace, spent coffee grounds, and almond shells, also deserves more attention because these materials may help regulate rumen lipid metabolism while supporting circular use of feed resources. However, their effects on biohydrogenation pathways, methane emissions, and product quality should be tested under controlled and species-specific conditions.

## 7. Conclusions

Rumen biohydrogenation plays a central role in determining the fatty acid composition of ruminant products. By transforming dietary unsaturated fatty acids into saturated products and bioactive intermediates, this process links rumen microbial metabolism with the nutritional quality of meat and milk. It is also connected with rumen hydrogen use and methane formation, although its direct contribution as a hydrogen sink appears limited.

Current evidence shows that rumen biohydrogenation can be influenced by diet composition, lipid source, plant secondary metabolites, rumen-protected lipid technologies, microbial interventions, and host-related factors. These strategies offer opportunities to improve the fatty acid profile of ruminant products, but their effects are often context-dependent and may differ among animal species, dietary conditions, and rumen microbial communities.

Several questions remain unresolved. The active microbes and enzymes responsible for many biohydrogenation steps are still incompletely characterized, and the biological significance of many transient intermediates requires further study. Future work should combine controlled feeding experiments with lipidomic and microbiome-based approaches to clarify causal mechanisms and support practical strategies for producing healthier and more sustainable ruminant products.

## Figures and Tables

**Table 1:** Microbial players of rumen biohydrogenation (BH).

Microbial Category	Microbial Function	Metabolic Process
*Butyrivibrio fibrisolvens*, *Pseudobutyrivibrio* spp.	Well-characterized group A bacteria involved in the early steps of rumen biohydrogenation [3]	Hydrogenate polyunsaturated fatty acids (PUFA) primarily into C18:1 (mainly trans-11 C18:1) [3,24]
*Butyrivibrio proteoclasticus*	The best-characterized cultured group B bacterium involved in terminal hydrogenation; highly sensitive to UFA toxicity [3]	Further reduce monoenes (mainly C18:1) into C18:0 [3,20]
Other candidate bacteria, including *Ruminococcus albus* and uncultured Lachnospiraceae- or Ruminococcaceae-related taxa	Some cultured strains can transform C18 PUFA into C18:1 intermediates, whereas many uncultured taxa are associated with BH phenotypes but remain functionally unvalidated [9,25]	These bacteria may participate in isomerization or partial hydrogenation steps; however, their species- and strain-specific roles require further validation [14,24]
*Cutibacterium acnes* (formerly *Propionibacterium acnes*)	Produces trans-10,cis-12 CLA in pure culture, although its quantitative contribution to the *in vivo* trans-10 shift remains uncertain [10,14]	Converts C18:2 into trans-10, cis-12 CLA, bypassing the normal trans-11 pathway [10,17]
Uncultured potential groups (WCHB1-41_ge, Lachnospiraceae, Rikenellaceae)	Uncultured taxa associated with BH extent, including WCHB1-41_ge and selected Lachnospiraceae- and Rikenellaceae-related taxa, have been identified through sequencing and multi-omics analyses. However, their direct catalytic roles and complete BH pathway capacities have not been experimentally validated [22]	These taxa are associated with BH extent and the formation of related intermediates, but their specific catalytic functions and positions within BH pathways remain to be experimentally validated [22]
Anaerobic fungi (*Orpinomyces*, *Piromyces*, *Neocallimastix*)	Capable of independent BH, though their metabolic rate is limited by a long life cycle (~24 h) [3,24]	Able to hydrogenate PUFA, but the metabolic products typically stop at the MUFA stage (e.g., C18:1) [24]
Protozoa (Ciliate Protozoa)	Do not synthesize BH enzymes; mainly act as reservoirs of lipids and associated BH intermediates [3]	Accumulate high concentrations of CLA and VA through the ingestion and sequestration of plant chloroplasts and symbiotic BH bacteria [3]

Abbreviations: BH, biohydrogenation; CLA, conjugated linoleic acid; MUFA, monounsaturated fatty acids; PUFA, polyunsaturated fatty acids; UFA, unsaturated fatty acids; VA, vaccenic acid.

**Table 2:** Regulatory strategies for rumen biohydrogenation (BH).

Regulation Category	Mechanisms and Impact on BH
Dietary Structure and Composition	
Forage-to-Concentrate (F:C) Ratio	High-forage diets generally favor the trans-11 pathway, whereas high-starch or high-concentrate diets, particularly when combined with a high rumen unsaturated fatty acid load, increase the risk of a trans-10 shift and the accumulation of trans-10 intermediates associated with milk fat depression [10,14,17]
Agro-industrial By-products	Partial replacement of cereal grains with pectin- or fiber-rich by-products, such as citrus pulp and almond hulls, can reduce dietary starch load and modify rumen fermentation, methane emissions, and the fatty acid composition of meat or milk. These changes may indirectly influence rumen BH, although direct evidence for consistent prevention of the trans-10 shift or improvement of BH completeness remains limited [14,26,27]
Lipid Supplementation	
Unsaturated Vegetable Oils	PUFA-rich vegetable oils increase the supply of substrates for rumen BH and may redirect a small proportion of metabolic hydrogen away from methanogenesis. In one goat study, corn oil supplementation reduced methane yield by approximately 15.1% per kilogram of dry matter intake, although the response depends on oil type, dose, basal diet, and animal species [4,28]
Marine Lipids and Microalgae	Marine lipids rich in EPA and DHA can inhibit the terminal reduction of trans-C18:1 intermediates to C18:0, thereby increasing the accumulation of trans-C18:1 and CLA-related intermediates. The magnitude and pattern of these effects differ between EPA and DHA and may vary among ruminant species [15,19,29]
Plant Secondary Metabolites	
Tannins (Condensed and Hydrolysable)	Condensed and hydrolysable tannins, including tannins from quebracho and *Terminalia chebula*, can alter rumen microbial populations and inhibit selected steps of BH, particularly the terminal conversion of trans-C18:1 intermediates to C18:0. Their effects on VA, CLA, and PUFA accumulation depend strongly on tannin source, dose, and dietary conditions [20,21,30]
Essential Oils	Essential-oil compounds such as cinnamaldehyde, carvacrol, and thymol can modify rumen microbial activity and alter the formation of BH intermediates. Their effects on PUFA disappearance and the fatty acid composition of meat or milk are dose- and diet-dependent [20,21,23,31]
Polyphenol Oxidase (PPO)	Polyphenol oxidase-rich forages, such as red clover, can reduce the extent of lipolysis and thereby limit the release of free unsaturated fatty acids available for subsequent rumen BH [9]
Chemical Additives and Stabilizers	
Ionophores (e.g., Monensin)	Monensin can alter the rumen bacterial community and modify PUFA biohydrogenation, resulting in changes in C18:2, CLA, and trans-C18:1 intermediates. However, the direction and magnitude of these responses depend on diet composition and experimental conditions [1,3,21]
Metabolic Modifiers	HMTBa supplementation has been shown to reduce trans-10 C18:1 concentrations in rumen digesta and milk fat and to maintain or increase milk fat concentration under diets with an elevated risk of biohydrogenation-induced milk fat depression. Responses may depend on production level and dietary risk factors [32,33] Potassium carbonate supplementation may modify ruminal BH and reduce the concentrations of selected trans fatty acids in milk while increasing milk fat concentration under some high-concentrate dietary conditions. However, effects on milk fat yield and the underlying mechanism are not consistent among studies [34,35]
Bypass and Protection Technologies	
Physical/Chemical Encapsulation	Rumen-protection technologies, including calcium salts, protein- or polymer-based encapsulation, and fatty acid amides, reduce the exposure of unsaturated fatty acids to rumen microbes and may increase their post-ruminal delivery. Protection efficiency varies with lipid source, processing method, and rumen conditions [1,9]
Natural Protection	Freeze-drying of *Nannochloropsis oceanica* preserves cellular integrity and partially protects EPA against ruminal metabolism, thereby increasing the potential post-ruminal availability of EPA [13]
Genetics and Microbiology	
Microbial Interventions	Selected microbial interventions have been investigated as potential tools for modifying rumen fermentation and BH. However, their effects on VA, CLA, rumen pH, fermentation efficiency, and animal performance are strain-, diet-, and host-dependent, and consistent benefits have not yet been demonstrated across studies [3,9]
Microbiome-driven Breeding	Host genomic factors influence approximately 27.6% of core rumen microbial genes. Selection for heritable microbial features may therefore contribute to improved meat fatty acid profiles and lower methane emissions, although validation across populations, diets, and production systems is still required [5]

Abbreviations: BH, biohydrogenation; CLA, conjugated linoleic acid; DHA, docosahexaenoic acid; EPA, eicosapentaenoic acid; F:C, forage-to-concentrate ratio; HMTBa, 2-hydroxy-4-(methylthio)butanoic acid; PPO, polyphenol oxidase; PUFA, polyunsaturated fatty acids; VA, vaccenic acid.

## Data Availability

No new experimental data were generated in this review. The data used for the figures and tables were obtained from published sources cited in the article and are available within the article and the Supplementary Materials.

## Supplementary Materials

The following supporting information can be downloaded at: https://www.mdpi.com/article/10.3390/ani16152424/s1, Table S1A: Paired fatty acid composition data and summary statistics used to generate Figure 1A; Table S1B: Paired feed and rumen-content unsaturation index values used to generate Figure 1B; Table S2a: Occurrence frequencies of standardized feed ingredients used to generate the word cloud in Figure 2A; Table S2bA: Feed-ingredient data used for hierarchical clustering in Figure 2B; Table S2bB: Fatty acid composition data used to generate the 2D pie charts in Figure 2C; Table S3a: Annual numbers of rumen biohydrogenation-related records involving dairy cows, meat sheep, and meat goats from 2014 to 2025; Table S3b: Flow values used to generate the Sankey diagram in Figure 4B.
