# Supplementary material for: A Review of Rumen Biohydrogenation and Efficient, Green Production of High-Quality Ruminant Products: Mechanisms, Opportunities and Challenges
Source: Animals (Basel). 2026 Aug 5;16(15):2424. doi: 10.3390/ani16152424 (PMC13464031; doi:10.3390/ani16152424)
Supplement: Supplementary file 1 [file animals-16-02424-s001.zip › animals-4419144-supplementary.pdf]

Table S1A. Paired fatty acid composition data and summary statistics used to generate Figure 1A.

| Dataset | Sample | C14:0 | C16:0 | C18:0 | cis-9 C18:1 | cis-9,cis-12 C18:2 | cis-9,cis-12,cis-15 C18:3 | Reference |
|---|---|---|---|---|---|---|---|---|
| 1 | Feed | 0.00 | 20.20 | 2.57 | 21.80 | 48.70 | 2.73 | [4] |
| 1 | Rumen contents | 5.78 | 30.10 | 33.30 | 7.16 | 7.68 | 0.58 | [4] |
| 2 | Feed | 0.00 | 15.00 | 1.79 | 25.90 | 53.70 | 1.31 | [4] |
| 2 | Rumen contents | 2.65 | 24.60 | 47.20 | 5.26 | 4.24 | 0.22 | [4] |
| 3 | Feed | 0.30 | 22.20 | 1.30 | 14.60 | 44.20 | 13.80 | [6] |
| 3 | Rumen contents | 0.93 | 16.16 | 33.08 | 3.03 | 2.57 | 0.29 | [6] |
| 4 | Feed | 0.70 | 22.50 | 3.10 | 18.20 | 40.30 | 12.60 | [6] |
| 4 | Rumen contents | 0.64 | 12.88 | 30.89 | 2.24 | 1.86 | 0.20 | [6] |
| 5 | Feed | 1.75 | 25.40 | 5.20 | 14.20 | 25.80 | 4.16 | [8] |
| 5 | Rumen contents | 3.20 | 26.10 | 32.90 | 5.38 | 2.94 | 0.07 | [8] |
| 6 | Feed | 0.00 | 15.30 | 5.10 | 71.00 | 20.70 | 3.30 | [7] |
| 6 | Rumen contents | 0.54 | 17.70 | 5.10 | 56.30 | 9.30 | 0.60 | [7] |

Notes: Fatty acid values are expressed as percentages of total fatty acids. Feed and rumen-content values with the same dataset number were obtained from the same publication. Reference numbers correspond to those in the main manuscript.

Summary statistics for Figure 1A

| Fatty acid | Feed mean | Feed SD | Rumen-content mean | Rumen-content SD |
|---|---|---|---|---|
| C14:0 | 0.46 | 0.69 | 2.29 | 2.04 |
| C16:0 | 20.10 | 4.18 | 21.26 | 6.66 |
| C18:0 | 3.18 | 1.65 | 30.41 | 13.74 |
| cis-9 C18:1 | 27.62 | 21.71 | 13.23 | 21.17 |
| cis-9,cis-12 C18:2 | 38.90 | 13.02 | 4.77 | 3.03 |
| cis-9,cis-12,cis-15 C18:3 | 6.32 | 5.43 | 0.33 | 0.22 |

Notes: Means and standard deviations were calculated across six paired datasets without weighting. No inferential statistical analysis was performed.

Table S1B. Paired feed and rumen-content unsaturation index values used to generate Figure 1B.

| | Description of the Diet | unsaturation index | Reference |
|---|---|---|---|
| Feed Formula 1 | Basal diet ( rice straw + concentrate+soybean meal + corn grain + wheat bran +$CaCO_3$+$CaHPO_4$ +NaCl+Premix ）; | 128 | 4 |
| Rumen Contents 1 | 10.5% CP, 46.8% NDF, 24.5% Starch ， 27.1% ADF， | 42.32 | |
| Feed Formula 2 | Basal diet ( rice straw +soybean meal + corn grain + wheat bran + $CaCO_3$ +$CaHPO_4$+NaCl+Premix+ 30 g/kg | 137.83 | |
| Rumen Contents 2 | DM corn oil） 9.99% CP, 45.0% NDF, 22.6% Starch ， 26.2% ADF， | 30.5 | |
| Feed Formula 3 | Barley,Soybean meal,Dehydrated alfalfa,Wheat bran 88.9%DM,18.0%CP,34.6%NDF,4.5%Ash | 145 | 6 |
| Rumen Contents 3 | | 83.92 | |
| Feed Formula 4 | Barley,Soybean meal,Dehydrated alfalfa,Wheat bran,Dried citrus pulp | 137.2 | |
| Rumen Contents 4 | 89.3%DM,18.5%CP,31.8%NDF,6.1%Ash | 123.8 | |
| Feed Formula 5 | Grass silage,Maize silage,Beet pulp,Brewer’s grains,Wheat,Extracted rapeseed meal,Extracted soybean | 94.48 | 8 |
| Rumen Contents 5 | meal | 36.81 | |
| Feed Formula 6 | Cracked corn grain,Barley grain,Soybean meal,Lucerne hay,Beet pulp,Molasses,Sodium bicarbonate,Mineral | 99.66 | 7 |
| Rumen Contents 6 | vitamin premix | 69.78 | |

Reference numbers correspond to those in the main manuscript.

Table S2a. Occurrence frequencies of standardized feed ingredients used to generate the word cloud in Figure 2A.

| Standardized feed ingredient | Occurrence frequency (n) | Standardized feed ingredient | Occurrence frequency (n) |
|---|---|---|---|
| Corn | 31 | Ammonium chloride | 1 |
| Corn silage | 27 | Bean pod hulls | 1 |
| Soybean meal | 26 | Berseem clover | 1 |
| Alfalfa | 21 | Cactus fruit peel | 1 |
| Cottonseed meal | 12 | Cactus fruit peel, pulp, and seeds | 1 |
| Wheat | 12 | Calcium ammonium nitrate | 1 |
| Beet pulp | 11 | Calcium salts of soybean oil fatty acids | 1 |
| DDGs | 11 | Corn bran | 1 |
| Barley | 10 | Cucumber straw | 1 |
| Rapeseed meal | 10 | Cucumber straw silage | 1 |
| Wheat bran | 10 | Dairy by-products | 1 |
| Barley silage | 5 | Expanded soybean meal | 1 |
| Grass silage | 5 | Fennel residue | 1 |
| Sunflower kernel meal | 5 | Freeze-dried garlic | 1 |
| Alfalfa haylage | 4 | Garlic | 1 |
| Dried beet pulp | 4 | Garlic extract | 1 |
| Commercial energy supplement | 3 | Heat-treated soybean meal | 1 |
| Oats | 3 | Hybrid rye silage | 1 |
| Tall fescue hay | 3 | Licorice straw | 1 |
| Biochar | 2 | Mannan oligosaccharides | 1 |
| Cereal bran | 2 | Oat hay | 1 |
| Grape pomace | 2 | Pellet binder | 1 |
| Grass hay | 2 | Perennial ryegrass hay | 1 |
| Monensin | 2 | Perennial ryegrass silage | 1 |
| Peanut haulm | 2 | Pistachio hulls | 1 |
| Peanut meal | 2 | Potassium chloride | 1 |
| Rumen-protected lysine | 2 | Rapeseed cake | 1 |
| Ryegrass hay | 2 | Rumensin | 1 |
| Soybean hulls | 2 | Tylosin phosphate | 1 |
| Spent coffee grounds | 2 | Wheat hay | 1 |
| Whole cottonseed | 2 | Wheat silage | 1 |
| Yeast fermentation product | 2 | White clover hay | 1 |
| Almond hulls | 1 | Yellow grease | 1 |

Note: Occurrence frequency represents the number of appearances in the compiled ingredient-frequency dataset. Ingredient names were standardized for spelling, capitalization, and spacing before counting. Word size in Figure 2A is proportional to occurrence frequency.

Table S2bA. Feed-ingredient data used for hierarchical clustering in Figure 2B.

| Feed ingredient | EE (%) | C14:0 | C12:0 | C16:0 | C16:1 | C18:0 | C18:1 | C18:2 | C18:3 |
|---|---|---|---|---|---|---|---|---|---|
| Corn | 3.60 | 0.10 | — | 11.10 | 0.40 | 1.80 | 26.90 | 56.50 | 1.00 |
| Sorghum | 3.40 | 0.20 | — | 13.50 | 3.20 | 2.30 | 33.30 | 33.80 | 2.60 |
| Wheat | 1.70 | 0.10 | — | 17.80 | 0.40 | 0.80 | 15.20 | 56.40 | 5.90 |
| Soybean meal | 1.90 | 0.10 | — | 10.50 | 0.20 | 3.80 | 21.70 | 53.10 | 7.40 |
| Cottonseed meal | 0.50 | 0.90 | 0.50 | 23.00 | 0.90 | 2.40 | 17.20 | 52.30 | 0.20 |
| Alfalfa meal | 2.30 | 1.90 | 2.00 | 25.60 | 1.40 | 3.80 | 4.40 | 19.30 | 37.00 |
| Soybean oil | 98.00 | 0.10 | 0.10 | 10.80 | — | 3.90 | 22.80 | 53.70 | 8.20 |
| Rice bran | 16.50 | 0.70 | 0.10 | 18.10 | 0.30 | 1.90 | 40.20 | 35.90 | 1.50 |
| Barley | 1.70 | 1.20 | — | 22.20 | — | 1.50 | 12.00 | 55.40 | 5.60 |
| Ground barley | 2.60 | 0.32 | — | 22.97 | — | 1.53 | 13.54 | 55.93 | 4.34 |
| Whole barley | 2.20 | 0.32 | — | 22.97 | — | 1.53 | 13.54 | 55.93 | 4.34 |
| Sugar beet pulp pellets | 1.40 | 0.14 | — | 26.66 | 0.39 | 0.89 | 11.55 | 49.83 | 6.35 |
| Chopped beet pulp | 1.40 | 0.14 | — | 26.66 | 0.39 | 0.89 | 11.55 | 49.83 | 6.35 |
| Full-fat rice bran | 13.40 | 0.21 | — | 17.53 | 1.46 | 1.56 | 39.10 | 38.11 | 1.46 |
| Soybean cake | 15.50 | 0.07 | — | 11.55 | 1.42 | 3.71 | 18.13 | 54.77 | 9.52 |
| Coarse-quality corn silage, DM 25%, NDF 41% | 3.20 | 0.31 | 0.36 | 17.83 | 0.36 | 2.42 | 19.24 | 47.74 | 8.25 |
| Medium-quality corn silage, DM 25%, NDF 41% | 3.20 | 0.31 | 0.36 | 17.83 | 0.36 | 2.42 | 19.24 | 47.74 | 8.25 |
| Coarse-quality corn silage, DM 25%, NDF 45% | 3.20 | 0.31 | 0.36 | 17.83 | 0.36 | 2.42 | 19.24 | 47.74 | 8.25 |
| Medium-quality corn silage, DM 25%, NDF 45% | 3.20 | 0.31 | 0.36 | 17.83 | 0.36 | 2.42 | 19.24 | 47.74 | 8.25 |
| Coarse-quality corn silage, DM 30%, NDF 49% | 3.20 | 0.31 | 0.36 | 17.83 | 0.36 | 2.42 | 19.24 | 47.74 | 8.25 |
| Medium-quality corn silage, DM 30%, NDF 49% | 3.20 | 0.31 | 0.36 | 17.83 | 0.36 | 2.42 | 19.24 | 47.74 | 8.25 |
| Coarse-quality corn silage, DM 40%, NDF 41% | 3.20 | 0.31 | 0.36 | 17.83 | 0.36 | 2.42 | 19.24 | 47.74 | 8.25 |

| Feed ingredient | EE (%) | C14:0 | C12:0 | C16:0 | C16:1 | C18:0 | C18:1 | C18:2 | C18:3 |
|---|---|---|---|---|---|---|---|---|---|
| Medium-quality corn silage, DM 40%, NDF 41% | 3.20 | 0.31 | 0.36 | 17.83 | 0.36 | 2.42 | 19.24 | 47.74 | 8.25 |
| Wheat bran | 4.50 | 0.10 | — | 17.05 | — | 1.08 | 17.81 | 59.09 | 4.73 |
| DDGs | 9.60 | 0.73 | 0.14 | 26.69 | 0.20 | 2.24 | 14.60 | 48.87 | 4.57 |
| Sunflower meal, CP 26% | 2.60 | — | — | 11.59 | — | 4.37 | — | 38.71 | 0.59 |
| Barley silage, CP 14%, NDF 50%, LNDF 8% | 4.00 | 5.50 | 0.80 | 43.40 | — | 4.10 | 7.30 | 12.30 | — |

Notes: EE is expressed as % of feed dry matter, whereas fatty acids are expressed as % of total fatty acids. A dash indicates that the corresponding value was not reported in the source literature and therefore could not be extracted; it does not represent zero. Missing entries were retained as missing values and were not manually imputed. Hierarchical clustering was performed in JMP Pro 16 using EE and eight fatty acid variables (C12:0, C14:0, C16:0, C16:1, C18:0, C18:1, C18:2, and C18:3). Variables included in the analysis were standardized before clustering; Ward's method was applied, the dendrogram was displayed on a distance scale, and three clusters were retained.

Table S2bB. Fatty acid composition data used to generate the 2D pie charts in Figure 2C.

| Fatty acid | Cereal grains (%) | By-products (%) | Forages (corn silage) (%) | Protein feeds (%) |
|---|---|---|---|---|
| C14:0 | 0.471 | 1.278 | 0.278 | 0.310 |
| C12:0 | 0.080 | 0.483 | 0.020 | 0.360 |
| C16:0 | 20.473 | 18.907 | 20.490 | 17.830 |
| C16:1 | 0.238 | 0.503 | 1.148 | 0.360 |
| C18:0 | 1.610 | 3.947 | 1.508 | 2.420 |
| C18:1 | 16.349 | 12.388 | 27.140 | 19.240 |
| C18:2 | 55.053 | 38.647 | 41.494 | 47.740 |
| C18:3 | 3.835 | 10.452 | 3.652 | 8.250 |

Notes: Values are expressed as percentages of total fatty acids and were converted from the proportional values used in the original OriginPro 2026 worksheet by multiplying by 100. The four columns represent predefined descriptive feed categories and are independent of the three clusters shown in Figure 2B. Totals do not necessarily equal 100% because only the eight displayed fatty acids were included; the values were not renormalized. The 2D pie charts were generated using OriginPro 2026.

Table S3a. Annual numbers of rumen biohydrogenation-related records involving dairy cows, meat sheep, and meat goats from 2014 to 2025, used to generate Figure 4A.

| Year | Dairy cows (n) | Meat sheep (n) | Meat goats (n) |
|---|---|---|---|
| 2014 | 31 | 12 | 9 |
| 2015 | 36 | 9 | 5 |
| 2016 | 42 | 20 | 10 |
| 2017 | 37 | 15 | 12 |
| 2018 | 48 | 19 | 12 |
| 2019 | 34 | 24 | 17 |
| 2020 | 37 | 14 | 10 |
| 2021 | 31 | 17 | 9 |
| 2022 | 38 | 24 | 12 |
| 2023 | 24 | 20 | 7 |
| 2024 | 33 | 16 | 8 |
| 2025 | 25 | 14 | 5 |

Note: Values represent the annual numbers of records classified under each animal category in the compiled dataset used to generate Figure 4A. The grouped column chart was generated using OriginPro 2026.

Table S3b. Flow values used to generate the Sankey diagram in Figure 4B.

| Source journal node | Target animal category | Flow value |
|---|---|---|
| J. Dairy Sci. | Dairy cows | 31 |
| J. Dairy Sci. | Meat sheep | 12 |
| J. Dairy Sci. | Meat goats | 9 |
| Anim. Feed Sci. Technol. | Dairy cows | 36 |
| Anim. Feed Sci. Technol. | Meat sheep | 9 |
| Anim. Feed Sci. Technol. | Meat goats | 5 |
| Animals | Dairy cows | 42 |
| Animals | Meat sheep | 20 |
| Animals | Meat goats | 10 |
| J. Anim. Sci. | Dairy cows | 37 |
| J. Anim. Sci. | Meat sheep | 15 |
| J. Anim. Sci. | Meat goats | 12 |
| Animal | Dairy cows | 48 |
| Animal | Meat sheep | 48 |
| Animal | Meat goats | 12 |
| Front. Microbiol. | Dairy cows | 34 |
| Front. Microbiol. | Meat sheep | 24 |
| Front. Microbiol. | Meat goats | 17 |
| Small Rumin. Res. | Dairy cows | 37 |
| Small Rumin. Res. | Meat sheep | 14 |
| Small Rumin. Res. | Meat goats | 10 |
| Livest. Sci. | Dairy cows | 31 |
| Livest. Sci. | Meat sheep | 17 |
| Livest. Sci. | Meat goats | 9 |
| PLoS One | Dairy cows | 38 |
| PLoS One | Meat sheep | 24 |
| PLoS One | Meat goats | 12 |
| J. Agric. Food Chem. | Dairy cows | 24 |
| J. Agric. Food Chem. | Meat sheep | 20 |
| J. Agric. Food Chem. | Meat goats | 7 |
| J. Anim. Physiol. Anim. Nutr. | Dairy cows | 33 |
| J. Anim. Physiol. Anim. Nutr. | Meat sheep | 16 |
| J. Anim. Physiol. Anim. Nutr. | Meat goats | 8 |
| Anim. Prod. Sci. | Dairy cows | 25 |
| Anim. Prod. Sci. | Meat sheep | 14 |
| Anim. Prod. Sci. | Meat goats | 5 |

Note: The Sankey diagram in Figure 4B was generated in OriginPro 2026 using a three-column input table consisting of source journal node, target animal category, and flow value. Flow width is proportional to the corresponding input value.
